# Neural mechanisms of maladaptive risk decision-making across psychiatric disorders

**DOI:** 10.3389/fnbeh.2025.1637582

**Published:** 2025-07-31

**Authors:** Cancan Lin, Yuhui Wang, Wenjie Xia, Defu Zhang, Xvbo Wang, Yue Wang, Yuxin Du, Hao Yu, Shanling Ji

**Affiliations:** ^1^Institute of Mental Health, Jining Medical University, Jining, Shandong, China; ^2^Department of Psychiatry, Shandong Daizhuang Hospital, Jining, Shandong, China

**Keywords:** risk decision-making, frontostriatal circuits, computational psychiatry, transdiagnostic, neuromodulation

## Abstract

Risk decision-making is a fundamental cognitive process that involves distributed neural circuits, with impairments observed across various psychiatric conditions. This systematic review synthesizes current evidence on the neurobiological substrates underlying maladaptive risk processing, highlighting three key findings. First, frontostriatal dysregulation is identified as a central feature, characterized by prefrontal hypoactivation and striatal hyperreactivity, particularly prominent in bipolar disorder and addiction. Second, disorder-specific neural signatures are noted, such as insular dysfunction in anxiety disorders, ventral striatal blunting in depression, and orbitofrontal-insula decoupling in schizophrenia. Third, computational modeling reveals distinct alterations in risk sensitivity, loss aversion, and reward valuation parameters across different diagnostic categories. This review also evaluates principal assessment methodologies and therapeutic interventions. Future research should prioritize the integration of computational psychiatry with multimodal biomarkers to advance both theoretical models and clinical applications.

## 1 Introduction

Risk decision-making represents a fundamental cognitive process that involves evaluating potential outcomes and selecting actions under conditions of uncertainty (Mishra, 2014). This complex neurocognitive function engages distributed neural circuits, particularly frontostriatal pathways that integrate reward valuation, risk assessment, and cognitive control mechanisms (Vorhold, 2008). Across psychiatric disorders, maladaptive risk decision-making manifests as a transdiagnostic impairment that significantly impacts functional outcomes and treatment adherence (Buelow, 2020; Camchong et al., 2014; Muir-Cochrane et al., 2011).

Advanced research has elucidated the neurobiological substrates of risk processing, identifying key roles for prefrontal cortical regions, the anterior cingulate cortex, and subcortical structures (Fan et al., 2024; Floresco et al., 2018; Freels et al., 2019; Gourley et al., 2013). These neural systems dynamically interact to compute risk–reward tradeoffs, with dysfunction in these circuits contributing to the decision-making deficits observed in mood disorders, addiction, and psychotic illnesses. Recent advances in computational psychiatry have enabled more precise characterization of decision-making pathologies (Carbó-Valverde et al., 2025; Mouchabac et al., 2022; Shimizu et al., 2022). Recent advancements in neuroimaging and biomarker research have elucidated potential targets for therapeutic intervention, encompassing neuromodulation of specific cortical regions and pharmacological modulation of monoaminergic systems (Wang et al., 2022). However, critical gaps remain in understanding how these neural mechanisms interact with metabolic, hormonal, and inflammatory pathways to influence risk preferences.

This review synthesizes current knowledge on the neural basis of risk decision-making across major psychiatric disorders, with particular focus on disorder-specific and transdiagnostic neural signatures, novel assessment approaches integrating computational modeling and neuroimaging, evidence-based intervention strategies targeting identified neural mechanisms, and critical future directions for research and clinical translation. By examining risk processing through integrative neuroscience and clinical perspectives, we aim to advance the development of targeted interventions that address these functionally significant cognitive impairments in psychiatric populations.

## 2 Brain regions involved in risk decision-making

Risk decision-making is regulated by a comprehensive network of cortical and subcortical regions that collaboratively process risk–reward tradeoffs, evaluate outcomes, and guide behavioral choices. During risk assessment, there are synergistic interactions among the prefrontal cortex, responsible for goal orientation; the striatum, which facilitates habit formation; the amygdala, which assesses emotions; and the monoaminergic circuits, which regulate dopamine levels (Orsini et al., 2015).

### 2.1 Cerebral cortex

The dorsolateral prefrontal cortex (DLPFC) serves as the cognitive control hub for risk-based decisions. This region is involved in assessing potential outcomes and integrating risk-related information to guide behavior. Additionally, the DLPFC is linked to the modulation of risk preferences, enabling the balance between potential rewards and associated risks of different choices (Gathmann et al., 2014; Jin et al., 2024).

The orbitofrontal cortex (OFC) is also particularly crucial in encoding the value of different rewards and integrating this information to guide decision-making processes. Studies have shown that the OFC is involved in value-based decision-making by processing the value differences between options and influencing choice behavior (Setogawa et al., 2019). The role of OFC extends to modulating decision-making under conditions of uncertainty, where it interacts with other brain regions such as the dorsomedial striatum to facilitate economic decision-making (Gore et al., 2023). Furthermore, the OFC connections with the ventral striatum are essential for outcome-based decision-making, highlighting its role in evaluating and integrating reward information to influence behavior (Gourley et al., 2013). The animal study also demonstrates that neurons in the OFC are involved in the execution of risk-related decision-making tasks in rats (Constantinople et al., 2019a; Constantinople et al., 2019b).

The ventromedial prefrontal cortex (VMPFC) and the ventral striatum are critical neural regions implicated in the representation of value and reward processing. These areas play a vital role in encoding the subjective value of various options and are activated during decision-making processes involving potential gains or losses. Specifically, the VMPFC integrates information regarding the desirability of outcomes, thereby facilitating value-based decision-making (Brosch and Sander, 2013; Wang et al., 2022).

The anterior cingulate cortex (ACC) is a pivotal region involved in conflict monitoring and error detection. The ACC becomes active during decision-making processes that necessitate the evaluation of conflicting information or adjustments in behavior based on feedback. It contributes to the assessment of decision riskiness and participates in the dynamic interplay among different neural mechanisms that shape decision biases (Kolling et al., 2014; St Onge et al., 2012).

### 2.2 Subcortical structures

The insula constitutes a significant region associated with risk processing and emotional responses to uncertainty. It is involved in the anticipation of risk and the emotional evaluation of potential outcomes, with its activation being linked to the perception of risk and the emotional impact of decision-making under uncertainty (Purcell et al., 2021; Schmidt et al., 2024).

The amygdala and the nucleus accumbens are engaged in the emotional and motivational dimensions of risk-taking. The amygdala is involved in the processing of fear and anxiety, which can influence risk-averse behavior, whereas the nucleus accumbens is implicated in reward anticipation and the motivational drive to engage in risky pursuits (Seok et al., 2015; Stopper and Floresco, 2014).

The ventral striatum, particularly the nucleus accumbens (NAc), plays a pivotal role in risk-based decision-making by encoding the subjective value of potential rewards. Dopamine signaling within the NAc is crucial for modulating risk preferences, with phasic dopamine release in the NAc core being associated with individual differences in risk-taking behavior (Sugam et al., 2012). The involvement of ventral striatum in decision-making is further supported by its interaction with the OFC, where it contributes to the processing of reward magnitude and influences impulsive choices (Diekhof et al., 2012; Freels et al., 2019). The role of the ventral striatum in integrating reward-related information is also evident in its contribution to cue-guided decision-making, where it helps refine reward-seeking behavior by mitigating the allure of unlikely rewards (Floresco et al., 2018).

The caudate nucleus and putamen, components of the dorsal striatum, are also critical in decision-making processes. The caudate nucleus is involved in linking outcome information to actions, providing spatial representations that are modulated by the prospect of risky outcomes (Yanike and Ferrera, 2014). This region supports goal-directed behavior by integrating reward-related and action signals, which is essential for flexible decision-making (Fan et al., 2024). The putamen, on the other hand, is implicated in the initial acquisition of instrumental behaviors and works in conjunction with the caudate nucleus during the early consolidation of these behaviors (Brovelli et al., 2011). The interaction between the OFC and putamen is particularly important for reversal learning performance, suggesting that these regions collectively contribute to adaptive decision-making by modulating behavioral flexibility (Groman et al., 2013).

Overall, the DLPFC, ACC, OFC, ventral striatum, caudate nucleus, and putamen form a complex network that underlies risk-based decision-making. These regions work together to evaluate risks and rewards, adapt behavior based on changing contingencies, and ultimately guide decision-making in uncertain environments. Understanding the distinct yet interconnected roles of these brain regions provides valuable insights into the neural mechanisms underlying decision-making and has implications for addressing decision-making deficits in psychiatric disorders.

## 3 Transdiagnostic neural signatures in risk decision-making

Risk decision-making is modulated by various neural mechanisms observable across a spectrum of psychiatric disorders, underscoring the notion of transdiagnostic neural signatures. These signatures represent neural patterns or activities that are consistent across multiple disorders, indicating shared underlying neural pathways that influence risk-related decision-making behaviors.

### 3.1 Bipolar disorder

Patients with Bipolar Disorder type I (BD-I) often exhibit risky decision-making, which is intricately linked to impulsivity and aggressive behavior. A study found that BD-I patients had significantly lower risky adjusted pump scores compared to healthy controls, indicating a propensity for maladaptive risk-taking behaviors (Ji et al., 2025). The dEC-based predictive model was particularly effective in forecasting non-planning and motor impulsiveness, underscoring the potential of neuroimaging techniques in identifying individuals at risk for maladaptive decision-making in BD-I (Ji et al., 2025). Further exploration into the neural underpinnings of risk decision-making in BD-I reveals aberrant temporal variability in brain connectivity. Specifically, increased dynamics in certain brain lobes and decreased dynamics in frontal regions were observed, which are associated with impulsive symptoms. The left supramarginal gyrus (SMG) emerged as a potential therapeutic target, influencing affective symptoms and risky behaviors as measured by the Balloon Analog Risk Task (BART) (Ji et al., 2021). These findings suggest that altered brain connectivity dynamics contribute to the impulsivity observed in BD-I, providing a neurobiological basis for targeted interventions. Research shows that in bipolar disorder, there’s an imbalance between pursuing immediate rewards and higher-order goals, leading to risky decision-making. This is linked to increased activity in the ventral striatum and reduced dorsolateral prefrontal cortex activity, indicating a bias towards immediate rewards. These findings offer a neuroanatomical basis for impulsive decisions in bipolar disorder and suggest intervention targets to improve self-control (Mason et al., 2014).

### 3.2 Depressive disorder

Individuals with major depressive disorder (MDD) tend to take more risks than healthy individuals, influenced by age, region, and task type. This indicates that risky decision-making in MDD varies based on demographic and contextual factors. The Iowa Gambling Task (IGT) and BART reveal these differences, with MDD patients being more risk-seeking in IGT and more risk-averse in BART, underscoring the complexity of decision-making in depression (Wang et al., 2024).

Metabolic factors affect effort-based decision-making and alter neural circuitry in MDD. A study of MDD found that markers of high insulin resistance and hyperglycemia were linked to less physical effort for rewards (Gill et al., 2025). Computational modeling showed that insulin resistance and cholesterol independently increased effort discounting. These metabolic changes may influence neural circuits involved in reward processing and decision-making. MDD patients were also found to have reduced risk sensitivity in the ventral striatum, suggesting inefficient reward processing (Gao et al., 2021). Additionally, event-related potential studies reveal that these patients are hypersensitive to negative feedback, with larger feedback-related negativity components linked to depression severity and psychological pain (Fan et al., 2021). These findings highlight the impact of altered reward and punishment processing on decision-making deficits in MDD.

Cognitive and affective factors significantly contribute to decision-making impairments in MDD. Research indicates that those with MDD often display maladaptive decision-making, such as procrastination and buck-passing, due to dysfunctional metacognitive beliefs and poor executive function (Singh et al., 2023). Additionally, childhood trauma and poor emotion regulation are key predictors of risk and loss aversion in depression, highlighting the impact of early experiences and emotional strategies on decision-making in MDD (Huh et al., 2016).

The social aspect of decision-making in MDD is crucial. Studies show that MDD patients have impaired social decision-making, exhibiting less trust and different cooperative behaviors than healthy individuals (Wang et al., 2024). This dysfunction is linked to issues in the lateral prefrontal-striatal/limbic networks, affecting executive control and emotion regulation (Shao et al., 2015). These impairments may lead to broader social dysfunction in MDD, highlighting the importance of targeted therapeutic interventions.

### 3.3 Addiction disorders

Risk decision-making is a critical component in understanding addiction disorders, as it often underlies the maladaptive behaviors associated with substance use and other addictive behaviors (Ariesen et al., 2023; Chen et al., 2020; Kohno et al., 2014). For example, adults with alcohol use disorder (AUD) tend to take more risks, which may be attributed to deficits in both affective and deliberative decision-making processes (Ariesen et al., 2023). This impairment is evident across different substances, including alcohol, tobacco, cocaine, and opioids, suggesting a pervasive issue across substance types (Chen et al., 2020). Methamphetamine users exhibit heightened activation in the ventral striatum and reduced activation in the dorsolateral prefrontal cortex (rDLPFC), suggesting a bias toward reward-driven behavior over cognitive control. This imbalance in neural activation may contribute to the maladaptive decision-making observed in addiction, emphasizing the need for interventions that target these neural circuits to improve decision-making and reduce addictive behaviors (Kohno et al., 2014). The evidence underscores the complexity of decision-making impairments in addiction disorders, involving both cognitive and neurobiological factors. These impairments are not only a consequence of addiction but also play a crucial role in its development and maintenance.

### 3.4 Anxiety disorders

In individuals diagnosed with Generalized Anxiety Disorder (GAD), the ventromedial prefrontal cortex (vmPFC) and dorsolateral prefrontal cortex (dlPFC) are implicated in future-oriented cognitive processing and reward perception (Nejati et al., 2025). A study involving 29 adults with GAD, who engaged in the BART and the Delay Discounting Task while undergoing transcranial direct current stimulation (tDCS), demonstrated that varying stimulation conditions targeting the vmPFC and dlPFC influenced risk-taking behaviors and reward processing. All active stimulation conditions enhanced the rate of updating prevalence and risk-taking behaviors in the BART. Specifically, anodal stimulation of the dlPFC combined with cathodal stimulation of the vmPFC improved prior beliefs regarding the likelihood of explosion and resulted in a more consistent pattern of decision-making.

Additionally, research on anxious adolescents suggests that the neural mechanisms underlying risk-taking behavior may differ from those in their non-anxious counterparts (Baker et al., 2024). Anxious adolescents self-reported increased avoidance behaviors, yet demonstrated normative risk-taking in laboratory tasks. The neural mechanisms underlying avoidance varied according to anxiety levels; activation in the left inferior frontal gyrus (IFG) was associated with risk avoidance in adolescents with low anxiety and with risk-taking in anxious adolescents. Conversely, striatal connectivity was linked to risk avoidance in anxious adolescents and risk-taking in those with low anxiety.

### 3.5 Schizophrenia

In the context of schizophrenia, significant impairments in risk decision-making have been documented, with its neurobiological underpinnings receiving increasing scholarly attention. A functional magnetic resonance imaging (fMRI) study comparing individuals with schizophrenia to healthy controls during decision-making tasks involving risk and ambiguity revealed no significant differences in risk attitudes (Fujino et al., 2016). However, individuals with schizophrenia exhibited a significantly higher tendency to choose ambiguity compared to controls. Furthermore, unlike healthy controls, individuals with schizophrenia did not show increased activation of the left lateral orbitofrontal cortex during decision-making under ambiguity as opposed to risk, suggesting a diminished aversion to ambiguity. Individuals with schizophrenia were observed to exhibit heightened activation in the left anterior insula, putamen, and frontal sub-regions during reward outcomes in the BART performance, alongside a reduction in grey matter volume in the left anterior insula (Tikàsz et al., 2019). These findings imply that the compromised decision-making abilities observed in schizophrenia patients may be attributed to an overvaluation of outcome-related stimuli.

## 4 Assessment for risk decision-making

### 4.1 Neuroimaging

Neuroimaging studies have elucidated the pivotal role of the prefrontal cortex in risk-related decision-making processes (Vorhold, 2008). Employing multi-voxel pattern analysis (MVPA) with fMRI, researchers have demonstrated that specific brain activity patterns can forecast individual variability in risk preferences. Notably, regions implicated in the representation of value and risk, including the prefrontal cortex, are instrumental in distinguishing between certain and risky choices (Wang et al., 2022).

Notably, neuroimaging investigations have revealed disorder-specific alterations in risk processing across psychiatric populations. In schizophrenia spectrum disorders, fMRI studies demonstrate atypical neural responses to ambiguous versus risky decisions, characterized by diminished activation in the left lateral orbitofrontal cortex compared to healthy controls (Fujino et al., 2016). Furthermore, individuals with schizophrenia exhibit hyperactivation in subcortical reward circuitry, including the left anterior insula and putamen, when processing reward outcomes (Tikàsz et al., 2019). Complementary findings in MDD indicate blunted risk sensitivity within the ventral striatum, reflecting impaired reward valuation mechanisms (Gao et al., 2021).

These neuroimaging discoveries collectively underscore the profound influence of aberrant reward and punishment processing on decision-making impairments across psychiatric conditions. The identification of such neural markers not only advances our mechanistic understanding of maladaptive risk-taking behaviors but also holds promise for developing targeted neurobiologically-informed interventions. Future research directions should focus on integrating multimodal neuroimaging data with computational modeling approaches to further elucidate the dynamic neural computations underlying risk decision-making pathologies.

### 4.2 Cognitive assessment tools

The utilization of cognitive assessment tools is crucial for assessing decision-making abilities in psychiatric populations. The MacArthur Competence Assessment Tools (MacCAT) are extensively employed for this purpose. A meta-analysis of 10 studies utilizing the MacCAT in schizophrenia revealed that patients with schizophrenia exhibited significant deficits in decision-making capacity compared to healthy controls (Wang et al., 2017).

In a study examining children and adolescents hospitalized due to acute mental disorders, the MacArthur Competence Assessment Tool for Treatment (MacCAT-T) was employed to evaluate treatment decision-making capacity (TDMC) (Mandarelli et al., 2017). The findings revealed variability in TDMC within the sample; however, the overall scores were favorable, indicating that children and adolescents with severe mental disorders might possess the competence to consent to treatment. TDMC demonstrated a positive correlation with cognitive functioning and a negative correlation with excitement levels.

## 5 Therapeutic strategies

### 5.1 Pharmacological interventions

Pharmacological interventions targeting specific neural pathways offer promising therapeutic approaches for improving risk decision-making in psychiatric populations. Current evidence highlights potential pharmacological mechanisms as follows.

#### 5.1.1 Lithium’s neuroregulatory effects

Lithium demonstrates unique properties in stabilizing risk-related decision making, particularly in bipolar disorder, through its modulation of monoaminergic systems. The drug’s action on dopamine receptors shows differential effects - while D1 receptor activation promotes preference for uncertain high-reward options, D2 receptor modulation exhibits more nuanced regulatory effects (St Onge and Floresco, 2008). Lithium’s therapeutic benefits appear to stem from its ability to normalize reward sensitivity and cost–benefit analysis through simultaneous regulation of both dopaminergic and noradrenergic pathways (Montes et al., 2015). These systems interact complexly in prefrontal-striatal circuits (Simon et al., 2011), where lithium may restore balanced receptor expression and activity patterns during decision-making under uncertainty.

#### 5.1.2 Stress hormone modulation

Research demonstrates a significant impact of cortisol on risk assessment, with hydrocortisone administration shown to reduce risk-taking in gain-oriented scenarios - an effect likely mediated through attenuated reward processing (Metz et al., 2020). The anterior insular cortex has been identified as a critical neural hub that integrates stress effects on decision-making processes (Shi et al., 2023), while observed sex differences in risk preference related to estrogen levels highlight the potential for developing hormone-targeted therapeutic interventions.

While pharmacological interventions demonstrate therapeutic potential, their optimal clinical implementation necessitates a multifaceted approach incorporating: (1) integration with evidence-based non-pharmacological interventions, (2) improved characterization of clinically meaningful patient subgroups through biomarker profiling, (3) development and validation of standardized decision-making metrics for reliable outcome assessment, and (4) deeper investigation of dynamic neural-hormonal interactions in risk processing pathways. The inherent complexity of decision-making pathologies, spanning molecular to behavioral levels, highlights the critical need for ongoing translational research to develop personalized, mechanism-driven treatment strategies that concurrently target both neurological circuitry and systemic physiological factors.

### 5.2 Cognitive-behavioral therapies

Cognitive-behavioral therapies (CBT) have been investigated as a means to enhance decision-making in psychiatric patients. A systematic review of neuropsychological interventions targeting decision-making deficits in addiction revealed that Goal Management Training (GMT) and Contingency Management (CM), when combined with CBT, hold promise for modifying decision-making processes (Verdejo-García et al., 2018). Specifically, GMT was found to enhance reward-based decision-making, whereas CM, in conjunction with CBT, positively influenced delay discounting.

In the context of schizophrenia, a study examined the relationship between pre-therapy OFC grey matter volume (GMV), emotional decision-making, and the response to cognitive-behavioral therapy for psychosis (CBTp) (Premkumar et al., 2015). The findings indicated that a greater OFC GMV was associated with improvements in positive symptoms, particularly hallucinations and persecution. Additionally, a greater rightward OFC asymmetry was linked to improvements in several negative and general psychopathology symptoms, suggesting that the OFC may play a significant role in the effectiveness of CBT in treating schizophrenia.

### 5.3 Neuromodulation techniques

Emerging neuromodulation techniques present novel opportunities for enhancing risk-related decision-making processes. The tDCS has been investigated for its effects on risk-taking behavior. In a study involving 16 participants, it was observed that left cathodal-right anodal tDCS significantly decreased risk-taking behaviors in contexts requiring rapid decision-making (Cheng and Lee, 2015). This reduction in risk-taking, compared to sham stimulation, was correlated with both state and trait impulsivity, with more pronounced effects observed in individuals exhibiting higher impulsivity levels.

Additionally, theta burst stimulation (TBS), an accelerated patterned form of magnetic stimulation, has demonstrated promising results. A comparative evaluation of intermittent TBS (iTBS), 20 Hz stimulation, and sham stimulation on healthy controls performing risk decision-making tasks (the Game of Dice Task and the Risky Gains Task) revealed that both iTBS and 20 Hz stimulation enhanced the utilization of negative feedback in the Game of Dice Task (Wang et al., 2021). Furthermore, iTBS exhibited a more substantial effect in reducing risk following negative feedback in the Risky Gains Task, indicating its potential clinical utility in fostering rational decision-making.

## 6 Future directions

Future research on the neural mechanisms underlying risk-related decision-making should prioritize several key areas. It is imperative to delve deeper into the neural substrates associated with various forms of risk-related decision-making. This includes examining the interactions between prefrontal, striatal, limbic, and monoaminergic circuits in complex decision-making scenarios characterized by risk and uncertainty (Orsini et al., 2015). A comprehensive understanding of how these neural circuits interact in real-world contexts and how their dysfunction contributes to aberrant decision-making in mental disorders is essential.

Additionally, investigating the role of individual differences, such as personality traits and genetic predispositions, in risk-related decision-making represents a significant research avenue. For instance, existing studies indicate that individual variations in risk-taking tendencies influence the neural processing of risky and ambiguous decision-making during adolescence (Blankenstein et al., 2018). Further research is needed to elucidate how these individual differences interact with neural mechanisms and contribute to the development of mental disorders characterized by impaired decision-making.

Computational psychiatry has advanced in breaking down decision-making under risk into distinct cognitive constructs. Research indicates that recalling positive memories can reduce risk aversion and alter probability weighting, suggesting therapeutic potential for psychiatric disorders (Shimizu et al., 2022; Watarai et al., 2023). This underscores the value of combining psychological interventions with computational models to improve decision-making. Interdisciplinary approaches may lead to new interventions that correct cognitive biases.

Digital psychiatry revolutionizes mental health care by using digital tools to improve diagnosis, treatment, and patient outcomes. Digital phenotyping, highlighted in research, helps reduce cognitive biases in psychiatry by collecting real-time behavioral data through connected devices, offering an objective view of patient behaviors and symptoms (Mouchabac et al., 2022). The study of neuroanatomy and neuropsychology in digital financial decision-making shows that neuropsychological factors like sensitivity to punishment and negative urgency are strong predictors of risk-taking (Carbó-Valverde et al., 2025). These findings emphasize the need to understand neural mechanisms in decision-making, applicable to psychiatric contexts where risk assessment is vital. This highlights the potential of digital psychiatry and tools like digital phenotyping to transform risk decision-making by offering a deeper understanding of patient behaviors and their neural foundations.

## 7 Conclusion

This review highlights the neural mechanisms underlying maladaptive risk decision-making in psychiatric disorders. The findings underscore the critical role of frontostriatal circuitry dysfunction, particularly the imbalance between prefrontal cognitive control systems and subcortical reward processing regions, as a transdiagnostic feature of impaired risk assessment. Disorder-specific patterns emerge, including ventral striatal hypersensitivity in bipolar disorder, blunted reward responsiveness in depression, and altered ambiguity processing in schizophrenia. Future directions should focus on creating targeted therapies based on individual neural profiles, using computational psychiatry to understand diverse clinical symptoms, and applying neuroscience research in clinical trials. This approach underscores the need to shift from symptom-based to mechanism-driven frameworks for personalized treatment of decision-making impairments in psychiatric patients.
